# Ideal Agent System with Triplet States: Model Parameter Identification of Agent–Field Interaction

**DOI:** 10.3390/e25121666

**Published:** 2023-12-16

**Authors:** Christoph J. Börner, Ingo Hoffmann, John H. Stiebel

**Affiliations:** Financial Services, Faculty of Business Administration and Economics, Heinrich Heine University Düsseldorf, 40225 Düsseldorf, Germany

**Keywords:** agent system, canonical ensemble, entropy, partition function, risk assessment, utility function

## Abstract

On the capital market, price movements of stock corporations can be observed independent of overall market developments as a result of company-specific news, which suggests the occurrence of a sudden risk event. In recent years, numerous concepts from statistical physics have been transferred to econometrics to model these effects and other issues, e.g., in socioeconomics. Like other studies, we extend the approaches based on the “buy” and “sell” positions of agents (investors’ stance) with a third “hold” position. We develop the corresponding theory within the framework of the microcanonical and canonical ensembles for an ideal agent system and apply it to a capital market example. We thereby design a procedure to estimate the required model parameters from time series on the capital market. The aim is the appropriate modeling and the one-step-ahead assessment of the effect of a sudden risk event. From a one-step-ahead performance comparison with selected benchmark approaches, we infer that the model is well-specified and the model parameters are well determined.

## 1. Introduction

Investors in individual stocks are sometimes confronted with sudden risk events that can occur due to individual company information. In addition to leaked company-internal information or (ad hoc) mandatory stock market announcements, the price-moving news can also appear in the form of unanticipated scientific publications. A current example of the latter is the shares of the pharmaceutical company BioNTech SE (ISIN US09075V1026). Political decisions, scientific studies on the vaccine produced, news about novel SARS-CoV-2 variants, and ad hoc statements from management make the price suddenly rise or fall, depending on the intensity and content of the new information.

In the phase after the information has been disseminated, the prices of the securities concerned show a dynamic that is largely decoupled from the overall market. The question arises whether such dynamics can be modeled appropriately and whether the risk event, which is a strong price movement or price jump [1,2], can be assessed one step ahead. From a practical perspective, these assessments allow statements to be made about the price development depending on news that might occur. In this way, risk events can be hedged ex ante.

The main focus of this study is on the empirical calibration of a top-down Ising-based model for sudden risk events using the BioNTech SE share as an example. On the other hand, there are bottom-up agent-based model approaches that also face the unsolved problem of appropriate empirical parameter calibration [3]. When these models are applied and tested on real data, empirical calibration, and validation is an issue that is widely discussed in the literature on agent-based models [4,5,6,7,8,9,10]. In particular, there is a need to explore how the models can be used in capital market applications, given the difficulty of empirically calibrating the models or properly choosing values for the parameters [3]. Although we cannot solve the problem in a generalized way, we contribute by showing how to empirically measure the model parameters of a top-down three-state model so that it can be used in practical applications. We show not only that it is possible to parameterize such a model, but also how to do so, and that it has practical utility in the application of forecasting. Thereby, we might help build the bridge between both approaches because some of the identified parameter values could possibly be used in bottom-up approaches. In addition, we develop the basic design for a forecasting procedure and compare its performance to selected benchmark approaches.

The approaches from the field of statistical physics, which in recent years have increasingly found their way into research in econometrics, appear to be a suitable framework for modeling such problems of abrupt price movements and the corresponding risk. These observations in econometrics are reminiscent of phase transitions in dynamical systems due to changing external variables. In physics, similar processes have been studied extensively and successfully in theory and practice since the early 19th century [11,12,13,14], e.g., spontaneous magnetization of matter. The statistical description of agent systems in econometrics as an image of a many-body system considered in statistical physics has experienced a significant boom in recent years and emerged as a separate strand of literature that can be traced back to the “Sociodynamics” research by [15] and somewhat later to the “Sociophysics” framework of [16], among other contributions. For an overview of the econophysics literature, see, e.g., [3,17,18,19,20,21,22].

The further developed methods in econometrics have been applied to a wide range of problems and particularly targeted the effects of human interaction: decision-making, voting behavior, capital market developments, etc. See, for example [23], who modeled the tendency of investors to be influenced by the investment attitude of other traders, which led to regimes of bubbles and crashes. Ref. [24] found imitations in three different data sources: birth rates, sales of cell phones, and the decline of applause in concert halls. Ref. [25] study a model in which interaction terms are reassessed continuously in time as investors are able to learn from past experiences. Ref. [26] proposed a generic model for multiple-choice situations in the presence of herding and compared it with data from a music market experiment. Ref. [27] studied membership dynamics in the open source software community with a spin model. Ref. [28] studied a model in which interaction dynamics are mediated by asset prices as a global variable accessible to every agent. Ref. [29] studied the process of investors’ opinion formation, and [19] reviewed recent studies on decision models. Ref. [30] studied the volatility of financial time series with an Ising system, and [31] proposed a variation of the Ising model to study the characteristics of stock markets. Ref. [32] studied a three-state model and attempted to understand how social processes such as cooperation or organization happen (this list is not intended to be complete). Furthermore, in Appendix A, the correspondence table (Table A1) can be used to trace in detail which sources guided us in transferring the variables from physics to econometrics.

Depending on the application in physics, various thermodynamic potentials are defined in statistical physics on the basis of partition functions, and all thermodynamic relations are derived from the latter. The different thermodynamic potentials are linked to one another via Legendre transformations, and the value of all partition functions are determined by eigenvalues of a defined functional H (*phys*.: Hamiltonian), i.e., the microscopic structure of the system under consideration. Hence, the various alternatives for the description of the state of the system under consideration are linked to one another. Statements that are derived in one model frame must be consistently reflected in the other frames.

In econometrics, the canonical partition function *Z* and the free energy *F*—as the associated thermodynamic potential—are usually considered for agent systems with binary decisions: “buy” or “sell”; “follow” or “not follow”; and “elect” or “not elect” (*phys*.: Two-State Spin-Systems) c.f. [19,33,34] and the vast literature cited therein. Applied to the stock market, this model approach describes the macroscopic behavior of a system of *N* agents (investors) who, through their binary decisions to “sell” or “buy” a stock, influence the price of the stock, accounting for the overall market and a flow of information. If instead a particular share and the acting agents are viewed as an isolated, closed system, decoupled from the overall market, then the microcanonical partition function Ω has to be evaluated, and the associated thermodynamic potential is the entropy S of the system. In what follows, we add the latter consideration to the existing stream of literature in econometrics. In addition, we tie in with existing approaches that extend the two states “buy” and “sell” with a third state “hold”, such as [28,35,36,37,38,39] among others. The analysis of the microcanonical partition function then better reflects reality and allows for a deeper insight into the underlying dynamics, the derivation of further parameters for the description and classification of risks, and a suitable interpretation of the functional H.

It is often discussed that H is related to the utility function *U* known in economics [19,33,34]. However, the following question arises: which utility for whom? One possible interpretation is to understand the utility from the perspective of a market observer (market analyst, researcher, investor) and to measure it in monetary units. The task of the market observer is then to describe the state of the market and to assess the effects of new information: for example, with the aim of determining the parameters of a predictive model and assessing the potential risk.

We specifically consider the ideal case in which new information reaches all agents simultaneously and instantly in a very short time, and, thus, possible risk events suddenly occur. Interactions between the agents play a subordinate role, and the system is therefore regarded as an ideal agent system. This approach allows the basic model parameters to be determined through empirical analysis. If the empirical setup is appropriate, the model parameters are determined largely free of other disruptive influences. The calculated, basic model parameters remain the same in all extended model concepts within statistical physics such that they can serve as a starting point for further, more complex models; see, e.g., [19,33,34,40,41] for an overview.

The most important extension in such models is the additional consideration of coupled investor behaviors. Such dynamics components superpose on the ideal agent system studied here and complicate the simultaneous determination of all model parameters. The essential approach to determine the parameters appropriately is to choose special market phases in which one part of the dynamics dominates. In this paper, we focus on the part of the dynamics describing investor behavior depending on the news environment. Thus, appropriate market phases are sought to determine the model parameters of this part of the dynamics.

The examination of the procedure, and how one obtains the parameters, especially in the case of financial market problems, is not yet widespread in the literature. Experimental physicists design an experiment, conceive the experimental setup, and measure the temporal behavior of quantities of interest to determine the parameters of a theoretical model and investigate physical properties. This is more difficult to realize in econometrics. In capital market models, partial event sets can be split off from a large number of past events, described by, e.g., price developments and general conditions, which can be assigned to the phenomenon to be examined.

Therefore, in our contribution, on the one hand, we adhere to the extension of proven models by introducing the “hold” position, i.e., investors’ stance to do nothing or not to change an existing equity exposure, and on the other hand, we focus on the design of the method for determining the model parameters, define the experimental setup and illustrate the method using empirical examples. For the case of an ideal agent system, we propose a way to identify this partial event set and how to determine the model parameters based on it. In addition, we show how the estimated parameters can be used to set up a one-step-ahead forecast model to assess abrupt risk events. The news field flows into the model as an external state variable and shares both technical and economic proximity to sentiment in finance literature. Sentiment is used in behavioral finance to predict asset prices [42,43,44,45]. Therefore, with our approach, we also show an innovative method to use the sentiment scores generated from text analysis for prediction. We apply the forecast and show its performance compared to selected benchmark approaches and deduce that the model is well-specified and the model parameters well-determined. Furthermore, the ideal agent system is included as a basic model in all known model extensions. Thus, the model parameters play a fundamental role in all extensions. In this respect, our contribution in the economic context can be viewed as fundamental research on which further analysis can be built.

The remainder of the paper is structured as follows: Section 2 outlines the theoretical model and defines risk indicators to describe immanent risks. In Section 3, we describe the capital market data required to determine the external state variables. Using the external state variables, we can then estimate the model parameters. The model and key risk indicators can then be used to assess sudden risk events. We formulate the idea of a one-step-ahead forecast model and show the performance of the three-state model compared to basic benchmark approaches and a two-state model. The last Section 4 summarizes our findings, discusses the limitations of the concept, and presents some ideas on how to extend both the model and the method for further research topics.

## 2. Method

We focus on the appropriate experimental design for the empirical determination of the parameters of the thermodynamic model for describing sudden risk events in the stock market. To this end, we consider the idealized, interaction-free theoretical model in which investors react independently of one another to new information. The parameters determined in this way are fundamental and, in accordance with the internal consistency of the thermodynamic approach, remain the same even in more complex models, for example with interactions.

In the following, we use the usual notations for describing model access; c.f., e.g., [19,41]. Typically, investors are referred to as agents in generalized models. We consider a system of *N* stocks that can be traded by a collection of agents. The model parameters are determined in Section 3 for a single financial asset on the basis of selected realizations in the capital market that come closest to the ideal case elaborated here. This idealization is similar to the case considered in ([33], Section 6.1). Similarly, normalization also takes place here, assuming that one asset is traded by one agent. In practice, each investor will generally trade more than one stock per order. Thus, for the number of shares per trade per agent, M≥1 holds. The normalization above is an approximation and justified if the number of all stocks, typically $N\approx 10^{8}$ and higher (e.g., BioNTech: $N\approx 2.5\times 10^{8}$), is much larger than the individual traded position $M\approx 10^{2}$; see also the similar discussion of Weiss domains in statistical physics [13,46].

In our transaction-based approach, each share can be bought, held, or sold. With the “hold” position, we thus expand the alternative courses of action and, overall, expand the existing model framework to include problems in the financial market. The three options “buy”, “hold”, and “sell” are typically called states of an agent and refer to an investor’s attitude towards a stock, i.e., how he positions himself towards it, and not to the existing positions (past transactions) in his portfolio. This has the advantage that all reactions to a new piece of information are modeled, and, thus, an instantaneous trading potential can be derived for the next time step, c.f. Section 3.4.

The three states are distinguished by the discrete variable $s_{i}=\left(-1,\;0,\;+1\right)$ for each stock i=1,…,N. A new message B with a basic sentiment sign(B)=(−1, 0, +1)—indicating “bad”, “indifferent” and “good” news—and a strength B=|B| affects the agents and thus the shares and ultimately the events in the capital market. We concentrate on the effects that sudden new information B triggers, which ideally is available simultaneously to all market participants, e.g., via Bloomberg L.P., Thomson Reuters Corp. or other competitors aggregating financial and legal news. The processes by which the agents interact with one another and diffusion processes, including trends and delays [19] for the information, are not considered here and are reserved for subsequent research on model extensions.

In the model framework considered here, each agent can be assigned a parameter $\mu_{i}$ that evaluates the individual change $\epsilon_{i}$ of H depending on the action $s_{i}$ in the external information field B. Depending on the application, $\mu_{i}$ has different names in the literature: willingness to adopt, willingness to pay, or idiosyncratic judgment [19,25,31,41]. In many studies, μ is chosen to be the same for all agents. Distributions ρ(μ) of the parameter or individual settings $\mu_{i}$ are considered in special extensions and applications, e.g., in sociophysics [33,34,40,47]. In the ideal case considered here, we initially set the parameter μ to be the same for all agents and thus follow the main stream of the literature. The focus of our empirical analysis of time series from the financial market in Section 3 is on the appropriate definition of the experimental setup and the determination of parameter μ.

As [19] notes, the decision of the individual agent depends on personal preferences, risk aversion, and framework conditions and is made given an individual utility function. In these investigations, the parameter μ models the tendency of the binary decision for or against an investment.

For a market analyst who does not know the individual circumstances of each agent and studies the level of the overall market, another interpretation of μ comes to mind. For the analyst, the parameter evaluates the change in utility that is inherent in a decision that conforms to the message. Consider a simple descriptive model: If the company news is bad and the agent decides to sell the stock, he or she behaves in accordance with the news, and the utility of the analyst increases if μ>0. The utility can thus be formulated from the perspective of the market analyst and, due to the conformity of the observation, could be interpreted as a cost reduction in the preparation of forecasts. How much the utility changes is then ascertained with the absolute value of μ. In this interpretation, μ can be understood as a parameter that evaluates the conversion of new information into utility for the market analyst.

If all agents are considered, the change in the overall utility *U* is calculated depending on a functional H. This functional is pivotal in thermodynamics, where its minimum is considered for special thermodynamic systems. Therefore, when considering a maximum utility in econometrics, a change of sign must still be accounted for: U=−H [34]. The functional H can be formulated based on the individual change $\epsilon_{i}$ triggered by an agent *i*:

(1) $$\begin{array}{cc} H & =\sum_{i=1}^{N}\epsilon_{i}. \end{array}$$

For the individual change $\epsilon_{i}$, a new discrete variable $S_{i}$ is introduced, which maps the fact that the individual agent behaves in conformity with the basic sentiment on company news, S=+1, or does not, S=−1. In the present case, we consider a triplet state system, and the position “hold” is also accounted for with S=0, so that an indifferent investor attitude is included. The position “hold” causes a fraction 0<α<1 of the individual changes of the other two positions and is always rated positively regardless of the basic sentiment of the news B. For the individual changes, we set

(2) $$\begin{array}{cc} \epsilon_{i} & =-\mu BS_{i}+\alpha \mu B\left(1-S_{i}^{2}\right) \end{array}$$

with μ>0 and B=|B|. For $S_{i}=+1$, the agent conforms to the new message, e.g., “sell” in the case of “bad” company news, and $\epsilon_{i}$ is negative and decreases H, i.e., increases the overall utility for the analyst or similar entity trying to understand or explain the market. A non-conforming decision (“sell” on “positive” news) increases H and decreases utility because the position requires explanation and involves utility-decreasing effort. The hold position consistently increases H, since deviation from conformity creates tension that requires explanation. However, because the situation is not as difficult to explain as the non-conforming decision, the reduction in utility may be proportionately smaller. Therefore, we expect the value range for α to be restricted between 0 and 1. The functional H depends on the set $\left(S_{1},\ldots ,S_{N}\right)$ and is simply

(3) $$\begin{array}{cc} H(S_{1},\ldots ,S_{N}) & =-\mu B\left(\sum_{i=1}^{N}S_{i}-\alpha \left(1-S_{i}^{2}\right)\right)\\ & =\mu B\left(N_{-}+\alpha N_{0}-N_{+}\right). \end{array}$$

Here, $N_{+}$ and $N_{-}$ are the number of agents who conform and do not conform to the company messages, respectively, and $N_{0}$ is the number of agents in the “hold” position. The following applies to the occupation numbers of each state $0\leq N_{-},\;N_{0},\;N_{+}\leq N$ and $N=N_{-}+N_{0}+N_{+}$.

The basic idea is to use the methods of statistical physics to derive a statistical model for the configuration $\left(N_{-},\;N_{0},\;N_{+}\right)$ that accounts for other macroscopic variables in addition to a particular company news item and allows inferences to be made about the number of potential buyers or sellers. The latter can then be used to estimate the impact on the price *P* of a share.

### 2.1. Canonical Ensemble

In the canonical ensemble, a subsystem is considered in thermal contact with an overall system. The exchange quantity in physics is the temperature *T*. In this case, one has to calculate the canonical partition function *Z*. In finance, this means that a stock and its investors are viewed as a subsystem embedded in an overall market. One strand of the literature [31] assumes that in econometrics, the role of temperature can be assigned to volatility σ. Thus, the volatility represents the exchange quantity in finance, i.e., T=σ. We share this perspective and develop this approach further in Section 2.2. It is customary to employ the inverse volatility (*phys*.: inverse temperature) β=1kT in the equations with a suitably defined constant *k* [19,23,27,29]. In Section 2.2, we present a suitable interpretation of *k* in finance (*phys*.: Boltzmann constant).

In statistical physics, the canonical partition function is defined as the sum over all sets $\left(S_{1},\ldots ,S_{N}\right)$:

(4) $$\begin{array}{cc} Z(T,B,N) & =\sum_{\left(S_{1},\ldots ,S_{N}\right)}\exp\left(-\beta H(S_{1},\ldots ,S_{N})\right). \end{array}$$

In Equation (4), we have a sum over so-called Boltzmann factors to evaluate the partition function. In statistical physics, this approach leads to the Boltzmann–Gibbs distribution specifying the probabilities of discrete states [13,23]. There is a broad discussion in choice theory about the use of this approach in finance or generally in sociophysics or econophysics [23,34,40]. The discussion in choice theory starts from the very mathematically convenient logit rule. This rule is equivalent to the abovementioned Boltzmann–Gibbs distribution, so the well-known results of statistical physics can be used [19].

In the simple case of noninteracting agents with Hamiltonian Equation (3), in a first step Equation (4) leads to:

(5) $$\begin{array}{cc} Z(T,B,N) & ={\left[Z(T,B,1)\right]}^{N}. \end{array}$$

Thus the canonical partition function is simply connected to the partition function for one agent. The latter follows immediately for an agent that can be in three possible states given a certain company news item:

(6) $$\begin{array}{cc} Z(T,B,1) & =\exp(-\beta \mu B)+\exp(-\beta \alpha \mu B)+\exp(+\beta \mu B). \end{array}$$

Let x=βμB. Then, with the partition function Equation (5), the probabilities for each state S=(−1, 0, +1) of the agent are:

(7) p1=Prob(S=−1)=exp(−x)exp(−x)+exp(−αx)+exp(+x)p2=Prob(S=−0)=exp(−αx)exp(−x)+exp(−αx)+exp(+x)p3=Prob(S=+1)=exp(+x)exp(−x)+exp(−αx)+exp(+x)

If we consider *N* stocks, Equation (7) leads to the configuration:

(8) N−=exp(−x)exp(−x)+exp(−αx)+exp(+x)NN0−=exp(−αx)exp(−x)+exp(−αx)+exp(+x)NN+=exp(+x)exp(−x)+exp(−αx)+exp(+x)N

#### 2.1.1. Trade Potential

With Equation (8), the average trade potential follows immediately:

(9) $$\begin{array}{cc} {\bar{N}}_{\mathrm{pot}} & =\mathrm{sign}\left(B\right)\frac{1}{N}\left(N_{+}-N_{-}\right)\\ & =\mathrm{sign}\left(B\right)\frac{\exp(+x)-\exp(-x)}{\exp(-x)+\exp(-\alpha x)+\exp(+x)} \end{array}$$

The trade potential is the balance of buyers and sellers at the respective point in time and represents the variable influencing the price. Note that if all agents sell on bad news ${\bar{N}}_{\mathrm{pot}}=-1$, and if all agents buy on good news, ${\bar{N}}_{\mathrm{pot}}=+1$ holds. If B=0 or volatility becomes infinite, the trading potential is ${\bar{N}}_{\mathrm{pot}}=0$. This describes the case in which the overall system has no clear direction in terms of selling or buying.

At this point, the theoretical question arises, under which conditions does the three-state system change into a two-state system? Specifically, what is the relationship between the two models? If x<∞ and it is assumed that α→∞, then Equation (9) reduces to the well-known relation for a two-state system: $|{\bar{N}}_{\mathrm{pot}}|\propto \tanh\left(x\right)$, c.f., e.g., [13,19]. This describes the case, which is not considered further here, in which the position “hold” would cause an infinitely large, negative effect related to utility. In this case, the position “hold” would not be taken. The value of α is expected to be between zero and one, so this case will not occur.

Since x=1, i.e., kT=μB, marks a special point, we approximate the trade potential Equation (9) in an asymptotic expansion for large (T→0 resp. B→∞) and a Taylor series for small (T→∞ resp. B→0) values of *x*:

(10) $$\begin{array}{c} {\bar{N}}_{\mathrm{pot}}\left(x\right)=\mathrm{sign}\left(B\right)\left\{\begin{array}{cc} \frac{2}{3}x+\frac{2}{9}\alpha x^{2}-\frac{1}{27}\left(\alpha^{2}+3\right)x^{3}+O\left(x^{4}\right) & x\to 0\\ 1-2\left(\exp(-2x)-\exp(-4x)+\ldots \right) & x\to \infty \end{array}\right. \end{array}$$

**Figure 1:**
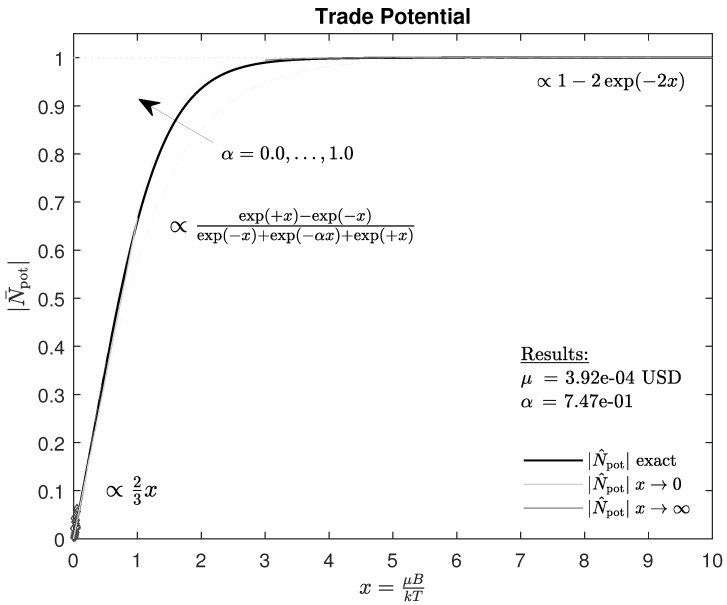
The average trade potential depending on *x*. The figure shows the exact function Equation (9) and the approximations Equation (10) for large and small *x*. Furthermore, the variation due to α is shown. All pairs of measured values (BkT,N¯pot) in Table 2 are used to fit the curve. The noted results (μ,α) correspond to the model parameters determined in Table 3 for the example under consideration.

If we consider the leading order in the first line, the dependence of the trade potential corresponds to the well-known law noted in [19], albeit with an extra factor of 23 that could have been guessed, because only 2 of 3 states are important for the trade potential. The extra factor was formally derived from theory and is important when estimating the true parameter μ. This is because if the estimation $\hat{\mu }$ of the parameter is based only on a two-state system (“buy” and “sell”), the result is likely to be a significant underestimation of the true parameter. Figure 1 shows the exact course of the trade potential, Equation (9), and its approximations, Equation (10).

#### 2.1.2. Utility

In a similar way, we can express the average utility in terms of x=μBkT. Substituting Equation (8) into Equation (3) leads to:

(11) U¯=−μBexp(−x)+αexp(−αx)−exp(+x)exp(−x)+αexp(−αx)+exp(+x).

The Taylor expansion for small *x* leads to the expression:

(12) $$\begin{array}{cc} \bar{U} & =-\mu B\;\left(\frac{\alpha }{3}-\frac{2}{9}\left(\alpha^{2}+3\right)x+\frac{1}{27}\alpha \left(\alpha^{2}-9\right)x^{2}+O\left(x^{3}\right)\right). \end{array}$$

For x=0, a basic negative average utility U¯=−μBα3 can be observed, which results from the fact that with high volatility and low message strength, the possible states are occupied equally by the agents and the utility of conforming and not conforming to the news offset one another.

#### 2.1.3. Risk Measures

For x≪1, i.e., the strength of the news B≈0 and a (constant) finite volatility, we can describe the behavior of the trade potential with small changes in the strength of the company message:

(13) $$\begin{array}{cc} \Delta {\bar{N}}_{\mathrm{pot}} & =\chi \Delta B\;\;\mathrm{with}\;\;\chi \left(T,B\right)=\frac{\partial {\bar{N}}_{\mathrm{pot}}}{\partial B}=\mathrm{sign}\left(B\right)\frac{2}{3}\frac{\mu }{k}\frac{1}{T}. \end{array}$$

The latter equation is the well-known Curie law χ∝1T noted in ([19], *phys*.: magnetic susceptibility) but with the extra factor 23.

Similar to Equation (13), it is also possible to approximate the change in the trade potential for small changes in volatility (*phys*.: thermal magnetic loss coefficient):

(14) $$\begin{array}{cc} \Delta {\bar{N}}_{\mathrm{pot}} & =\eta \Delta T\;\;\mathrm{with}\;\;\eta \left(T,B\right)=\frac{\partial {\bar{N}}_{\mathrm{pot}}}{\partial T}=-\mathrm{sign}\left(B\right)\frac{2}{3}\frac{\mu }{k}\frac{B}{T^{2}}=-\frac{2}{3}\frac{\mu }{k}\frac{B}{T^{2}}. \end{array}$$

In the same way, for x≪1, we can express changes in utility with a small change in volatility. Considering Equation (12) up to order O(x) and B=const., the change can be described as follows:

(15) $$\begin{array}{cc} \Delta \bar{U} & =c_{B}\Delta T\;\;\mathrm{with}\;\;c_{B}\left(T,B\right)=\frac{\partial \bar{U}}{\partial T}=-k\frac{2}{9}\left(\alpha^{2}+3\right){\left(\frac{\mu B}{kT}\right)}^{2}. \end{array}$$

The coefficient $c_{B}$ is a kind of capacity (*phys*.: specific heat capacity). It describes the ability of the agent system to react to changes in volatility in the form of changes in utility. Since $c_{B}$ is always negative, every increase in volatility leads to a loss of utility. With increasing volatility, however, the effect decreases, and the absolute change in utility becomes smaller. In the high volatility limit, T→∞, no change in utility can be determined.

Similar to the concept of duration, the coefficients χ, η and $c_{B}$ can be understood as risk parameters if there are sudden minor changes in the news situation or in the volatility: For small changes, they can be used to estimate the effect on the considered quantity (trade potential or utility).

### 2.2. Microcanonical Ensemble

The calculation and evaluation of the microcanonical partition function Ω leads to the entropy of the whole agent system [13,23]:

(16) $$\begin{array}{c} S=k\ln\Omega . \end{array}$$

Its similarity to Shannon’s term for information [48] suggests that entropy should be interpreted as the information deficit of the market analyst about the microstate, i.e., the state of a single agent, associated with knowing the macroscopic variables, e.g., B and *T*. The greater the entropy, the less the market analyst knows about the microscopic state and the less information he or she knows about the entire agent system. Shannon himself introduced *k* as a positive constant and assigned it the property of a unit of measurement ([48], p. 11). Considering the microcanonical partition function Ω in detail, we want to pursue this idea further to gain a suitable interpretation of the constant *k* in the finance field.

In finance, the partition function Ω counts the number of sets $\left(S_{1},\ldots ,S_{N}\right)$ that lead to the same, pre-given utility *U*. For the calculation, a small interval [U−δU,U] is defined, so that only some configurations $\left(N_{-},\;N_{0},\;N_{+}\right)$ according to Equation (3) lead to a utility within the interval. If the maximum entropy principle [33,49] is employed, all that remains is a single configuration that meets the utility specification. Then, the number Ω of possible sets that create the configuration is counted. In the present case, with δU<αμB, the number of sets is determined by the multinomial coefficient:

(17) $$\begin{array}{cc} \Omega & =\frac{N!}{N_{-}!N_{0}!N_{+}!}. \end{array}$$

Using Stirling’s formula, lnn!=nlnn−n, and norming with a factor 1ln3, the average entropy is

(18) $$\begin{array}{cc} \bar{S} & =-k\sum_{i=1}^{3}p_{i}\log_{3}p_{i} \end{array}$$

with the probabilities $p_{i}$ defined in Equation (7). If k=1, then $\bar{S}$ is the entropy in a ternary numeral system with minimum $\bar{S}=0$ and maximum $\bar{S}=1$. If pi=13, then the agents are evenly distributed over all possible positions (“sell”, “hold”, and “buy”). Then, the entropy is at its maximum, and a market analyst needs one trit (trinary digit, c.f. [50]) of information to determine the exact setting of an individual agent. A similar discussion is held for the two-state systems (“buy” and “sell”) with bits in [51]. An obvious interpretation would be to regard *k* as the cost of obtaining the information. The unit of measurement of *k* would then be monetary units. If $p_{i}=1$ for some *i*, then the entropy is zero, i.e., all agents are in the same state *i*, every microstate is thus the same, the market analyst does not need any further information to make statements about the position of an individual agent, and there are no costs for further information.

In this interpretation of *k*, the entropy of the entire agent system would sum up the cost of the uncertainty, i.e., the information deficit measured in trits. The constant *k* is, therefore, not a model parameter but defines the unit of measurement.

#### State Quantity *T*

In statistical physics, the temperature *T* is a state quantity that is independent of the amount of matter considered in a thermodynamic system. Analyzing the microcanonical ensemble, one finds a determining equation for the temperature. A similar approach was proposed by [34] to define the state quantity *T* in agent systems. We will briefly retrace the course.

According to its calculation, entropy S, Equation (16), is a function of utility *U* for a fixed number of agents *N* and given news strength *B*. If a Taylor expansion is implemented with respect to *U* and second- and higher-order terms are neglected, then [34] finds the following relationship in analogy to statistical physics:

(19) $$\begin{array}{cc} \frac{1}{T} & =-\frac{\partial S}{\partial U}{|}_{U} \end{array}$$

Since [34] neglects *k* in the entropy, he comes to the interpretation that in economics, the state quantity *T* can be considered as the price of (negative) entropy. Furthermore, he interprets that the parameter *T* plays the role of an *internal* temperature of an agent, and it measures its deviation from rational behavior, which is recovered as T→0. This can include, for example, not behaving in conformity with the company news B, i.e., buy on bad news, or changing positions quickly without any new information. For a stock price, this leads to an observable volatility. Furthermore, based on the observation that in many capital market-related applications, a proportionality between temperature and volatility is assumed T∝σ [19,52], it was recently shown for an Ising-based two-state model that there is a fundamental relationship between temperature and trading volatility [53]. Thus, in this interpretation, the volatility observable on the capital market is the measurand for determining the internal temperature, and in the simplest case, the measuring equation is T=σ. In the sense of the microcanonical view, a closed stock/agent system *has* a volatility. If the system is considered embedded in the overall market (canonical ensemble, Section 2.1), then the exchange quantity is the volatility, and the individual system *is* influenced by the volatility of the overall market.

The main qualitative effects of a changing temperature can also be observed in terms of volatility. If the volatility tends toward zero, decisions become easier even with weak new news and are primarily based on the direction of the new news. The agent system overall conforms to the company message (*phys*.: crystallization). If the volatility becomes very high, decisions with the same information become more difficult; the direction fluctuates and is not necessarily based on the new message. As in the physics of spin systems, the increasing disorder can be observed in the agent system, and no direction dominates. Ref. [16] conducted a similar discussion in sociology when interpreting his model of a strike, which is based on two states (“work state” and “strike state”).

If we continue to assume that the entropy and utility itself can be measured in monetary units, then *T* becomes a unitless quantity that mediates between small changes in entropy and changes in utility in the following way: ΔU∝−TΔS, i.e., an increase in entropy interpreted as costs for further information from the perspective of a market analyst decreases his or her utility, and the greater the volatility is, the stronger the effect.

There can be the unlikely situation that if the news is bad, the agents are predominantly in the “buy” position, or if the news is good, they are predominantly in the “sell” position. In both cases, $N_{+}-N_{-}<0$. For the trade potential, Equation (9), ${\bar{N}}_{\mathrm{pot}}>0$ (buyer surplus) is calculated for bad news and ${\bar{N}}_{\mathrm{pot}}<0$ (seller surplus) for good news. In any case, the entire agent system does not behave in accordance with the news situation.

Such an inversion is known in statistical physics and leads to a negative temperature being assigned to the inverse state. In general, these states are not stable, and the system relaxes within a short time [13].

The same is assumed for the agent system considered here. An inverse state, triggered, for example, by two successive company messages with rapidly changing signs, should relax within a short time. The transition from the “inverse” to the “normal” state generally takes place via a sequence of nonequilibrium states, and methods from nonequilibrium thermodynamics are to be used for modeling, c.f., e.g., the propagation of partition functions depicted in [11]. The just-described transition can only be modeled with the (suitably extended) methods described in this section if it is a sequence of quasi-steady state changes in the system.

In what follows, we are interested in a procedure to estimate the model parameters μ, α and examine the “normal” case, where the entire agent system behaves in accordance with the news situation. This means that we will not further consider the above-described case of an inversion of the agent system in the following.

## 3. Application

### 3.1. Procedure for Determining the Model Parameters—Experimental Setup

The model parameters 0<μ and 0<α<1 are to be determined on the basis of capital market data. The idealized model presented in Section 2 focuses on mapping special risk events for single financial assets. By construction, the model is not designed for the entire dynamic but only for special components of the dynamic in which the parameters μ and α essentially determine the dynamic. If there are temporary events in the capital market that correspond to the idealized specifications, the model parameters can be determined in this cut-out experiment. The following procedure is used to determine the parameters and takes the idealized conditions into account.

1. Periods of low, constant volatility *T* with sideways price movement are sought for the single asset to be examined. Quantile specifications are used to search for such time segments. In the ideal case, the time segments found correspond to dynamic equilibria.
2. The news situation is then examined for all the time segments found, and those time segments are selected for further analysis in which a single central message B dominates the following period. The strength B=|B| and direction sign(B) of the message are determined.
3. We set the value of *k* to one monetary unit, compare Section 2.2, and calculate BkT. This means that μ remains in x=μBkT as a variable that has yet to be determined.
4. Next, the seller or buyer surplus ${\bar{N}}_{\mathrm{pot}}$ is determined from the shares traded. There are several ways to estimate ${\bar{N}}_{\mathrm{pot}}$; we discuss three different approaches and suggest one for further use. A pair of measured values (BkT, N¯pot) is thus calculated for each examined time segment.
5. All pairs of measured values (BkT, N¯pot) are used to fit the curve shown in Figure 1 with Equation (9) and determine the model parameters μ and α.

### 3.2. Data

**Figure 2:**
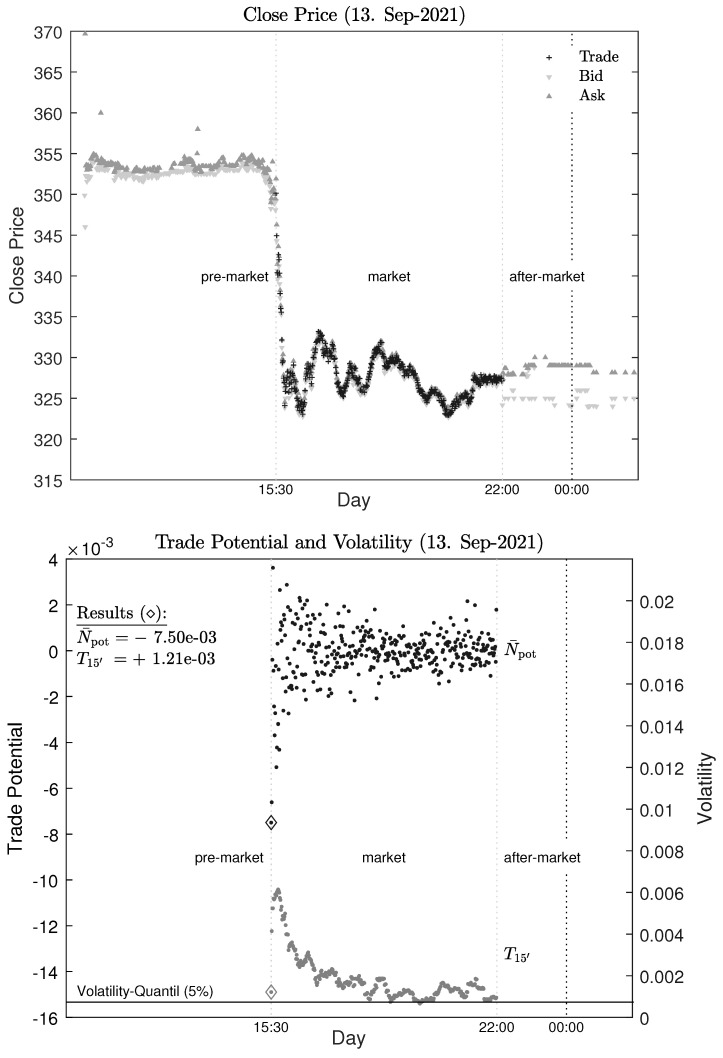
Sampled closing prices for BioNTech are in the upper panel. Trade potential and volatility are in the lower panel. Time scale: UTC+2.

As an example, the shares of the pharmaceutical company BioNTech are examined in detail. Bloomberg trading data for this company is available from the American Stock Exchange NASDAQ for the period from 1 August 2021 to 31 January 2022 with a sample rate of 1 min (Bloomberg code: BNTX UW). The dataset is analyzed for the market session and the pre- and after-market sessions. The opening, closing, high, and low prices are available for each minute. In addition, the sales volume, sales value, and number of ticks are attached to the price data. An excerpt in Figure 2 (upper panel) shows the closing prices of the various sessions divided into bid, ask and trade for 13 September 2021.

#### 3.2.1. Volatility *T*—Temperature

For the statistical model in Section 2, the external state variable *T* is required and must be extracted from the capital market data. The state variable *T* was interpreted as instantaneous volatility, which is the volatility that market participants observe for the share at the moment prior to becoming aware of the news B. This means that the volatility must be determined over a period that is as short as possible to be measured as closely as possible to the event and to include as few other effects as possible. The goal of volatility measured as close to an event as possible is thwarted by a lower limit of data points used. The limit is given by the fact that the statistical error is becoming more dominant. We successively examined shorter measurement periods of 120, 60, 30, 15, 10, and 5 min and found that below 15 min, the statistical errors dominate in determining the volatility. For the following analyses, the volatility, and, hence, the state variable *T*, are determined based on logarithmic returns over the shortest reasonable time window of 15 min. Thus, the volatility at each time *t* is determined continuously as the standard deviation over 15 logarithmic returns ($t,\;t_{-1},\ldots ,t_{-14}$) in a rolling window.

#### 3.2.2. Trade Potential ${\bar{N}}_{\mathrm{pot}}$—Magnetization

Another key variable required is the effective trading potential ${\bar{N}}_{\mathrm{pot}}$, which is calculated from the buyer surplus or seller surplus excluding hold positions, c.f. Equation (9). Different approaches were examined to extract the trade potential from the market data. The attempt to estimate trading potential based on the bid-ask difference in turnover volume failed because the bid and ask volumes also include some older stop-loss orders, stop-buy limit orders or other types of orders that are not necessarily related to the current event. A similar problem prevents the direct use of trading volume as a proxy for the trade potential a step back in time. In the case of a price jump, older limit specifications can be processed. As a result, the trade potential attributable to the actual event may be inaccurate. If only one stock exchange is examined, it is also unclear how *N* is to be set in Equation (9). Thus, with the data and especially the sales volume available to us, the trade potential cannot be determined with sufficient accuracy. Therefore, we use an indirect method to estimate the trade potential. Thereby, the concatenation factors Kt=Pt+1Pt of the instant price movement at a certain point in time *t* are calculated. Then, the trade potential is estimated following [28]:

(20) $$\begin{array}{cc} {\hat{\bar{N}}}_{\mathrm{pot}}\left(t\right) & =\frac{K_{t}-1}{K_{t}+1} \end{array}$$

With the limit $P_{t+1}\to \infty$, the trade potential tends to +1 (all agents buy on good news), and with $P_{t+1}\to 0$ the trade potential tends to −1 (all agents sell on bad news), given finite price $P_{t}>0$. With Equation (20), the following equation is determined for the estimated price at time t+1 given the price at time *t* and the trade potential defined in Equation (9):

(21) $$\begin{array}{cc} {\hat{P}}_{t+1} & =\frac{1+{\bar{N}}_{\mathrm{pot}}}{1-{\bar{N}}_{\mathrm{pot}}}P_{t}\;\mathrm{with}\;{\bar{N}}_{\mathrm{pot}}=\mathrm{sign}\left(B\right)\;\frac{N_{+}-N_{-}}{N}. \end{array}$$

An excerpt in Figure 2 (lower panel) shows the estimated trade potential and the volatility for 13 September 2021.

Ref. [28] offer the left part of Equation (21) as an approximated price function and find that the exact form is not critical to their results. One way to derive this equation is to find a price *p* of the asset in the time interval [t, t+1], from which the price $p_{t+1}=\exp\left({\bar{N}}_{\mathrm{pot}}\right)p\approx \left(1+{\bar{N}}_{\mathrm{pot}}\right)p$ is calculated in the forward direction and the price $p_{t}=\exp\left(-{\bar{N}}_{\mathrm{pot}}\right)p\approx \left(1-{\bar{N}}_{\mathrm{pot}}\right)p$ in the backward direction.

The relation $p^{\prime }=\exp\left({\bar{N}}_{\mathrm{pot}}\right)p$ is obtained for the expected path of the stochastic process described in ([54], Equation (5)) when ${\bar{N}}_{\mathrm{pot}}$ is constant for the time interval under consideration. If $|{\bar{N}}_{\mathrm{pot}}|\ll 1$, then $p^{\prime }=\left(1+{\bar{N}}_{\mathrm{pot}}\right)p$ holds approximately. If the evolution from $p_{t}$ to $p_{t+1}$ is performed over the intermediate price *p*, the equation proposed by [28] is calculated. This equation has the property of shape invariance under time reversal and is suitable to describe reversible processes, i.e., ${\bar{N}}_{\mathrm{pot}}\to -{\bar{N}}_{\mathrm{pot}}$, in the context of quasi-stationary state changes [13].

#### 3.2.3. News Sentiment B—The Magnetic Field

As described above, qualified messages must first be identified. These are characterized by the fact that the price development of the company share associated with them is caused exclusively and instantly by the news itself and is, therefore, largely independent of overall market developments. If this is the case, we are closest to the case of the ideal agent system under consideration.

A Bloomberg terminal is used to analyze the news situation. This allows us to view all news at specific points in time. To ensure that the events are not distorted by another influencing variable, such as a macroeconomic shock or similar factor, we check the volatility of the BioNTech share itself and the volatilities of two indices representative of the market (S&P 500 and NASDAQ-100) in the 15 min before the events.

The idea is that the financial market “system” must be in a tranquil state, representing a dynamic equilibrium, in each case before an event happens. In our view, the stock itself and the indices are each in dynamic equilibrium or in a state of tranquility if the range of volatility in the 15 min before the event is smaller than the 95% quantile of volatility in our overall time period (August 2021 to January 2022) and does not change In experimental physics, one would measure the following: The temperature of a spin-system is low and constant, and the entire system is in dynamic equilibrium with the thermal bath [11,13]. In other words, the market is in dynamic equilibrium if it is not subject to large fluctuations (negative delimitation).

Out of 24 potential events, there thus remains a sample of 18 carefully identified events, where each event is enriched by some splitter messages that make the same statement as the so-called lead headline, which is representative of the respective news package for an isolated event at a specific point in time. Once the qualified events are identified, the empirical challenge in determining B is to convert the qualitative information of a message into a quantitative measure. In doing so, the strength B=|B| on the one hand and the direction of the information sign(B) on the other hand must be determined.

We resort to a textual analysis based on a linguistic approach to evaluate text fragments and calculate sentiment scores. The approaches in the field of text analysis are manifold, and the different methods offer their individual advantages, c.f., e.g., [55,56,57]. In particular, two-dimensional approaches with “positive” and “negative” sentiments are effective at capturing different contexts (such as the economic context of business-related messages) or at dealing with linguistic peculiarities [58].

However, before the messages can be evaluated, a common problem in text analysis must be considered by preprocessing the data. Since inflected words can deviate from their root word, a matching algorithm might fail to correctly assign these variations to the root word. Therefore, the words in the message must first be transformed. Linguists have proposed several approaches to address this problem [59]. Stemming, for example, traces a word to its root by identifying and eliminating suffixes. Lemmatization, on the other hand, groups inflected words into a single group. We first transform our data using the lemmatization list (41,531 words) created by [60], which can be accessed via the R package “textstem” by [61]. In selecting a suitable linguistic approach, we included several well-known dictionaries that are embeddable via the R package “SentimentAnalysis” of [62]. These include Henry’s Financial Dictionary [63], the Harvard-IV dictionary, the Loughran–McDonald Financial Dictionary [64], and the QDAP dictionary from the R package “qdapDictionaries” by [65]. The algorithm additionally performs preprocessing operations such as removing stopwords and stemming. With each dictionary, lists of positive and negative words are used, and the occurrences in the messages are counted. The sentiment scores are the netted occurrences of positive, *P*, and negative, *N*, words, divided by the respective number of words *M* in a news package to control for messages of different lengths: $B=\frac{P-N}{M}$. According to this definition, news sentiment B is measured in fractions of unit one and takes values between −1 and +1.

A major drawback of the scoring algorithm is that it does not account for negations (i.e., words such as “not”), resulting in potentially misidentified scores and sentiments. For example, the algorithm would count the phrase “not successful” as +1 instead of −1. For this reason, we manually edit our data by multiplying the determined sentiment score of the words that are negated by −1. We refrain from further text transformation at this point, such as smiley recognition, since this level of sophistication is sufficient to analyze the mostly standardized text messages in finance.

The sensitivity of the sentiment analysis can be further increased if the dictionaries are supplemented with a few additional keywords appropriate to the problem. We motivate this measure with the conclusion and result of [44,55] that more field-specific dictionaries are needed. The addition of a few but salient keywords with high relevance in the pharmaceutical (and financial) context shows that this measure is already very effective, and there is no need to define a complete dictionary in the first approach. The supplement of suitable keywords to dictionaries through human intervention is an established procedure in machine translation. Human intervention can be found in the context of machine translation when editing results and when creating or adapting dictionaries. In practice, human intervention is used to increase the performance of machine translation [66]. The latter is driven by the fact that basic dictionaries may not be able to handle specific—industry standard—terminologies. Ref. [66] deduces an “add-and-delete” strategy since, on the one hand, important words should be added to the dictionary, and on the other hand, superfluous words that do not fit the context at hand should be removed to avoid creating erroneous ambiguities. In our context of the BioNTech stock, we keep, e.g., the basic financial context of the Loughran–McDonald dictionary but add (a few strictly selected) words from the pharmaceutical (and financial) context (if they are not already included in the respective dictionary): “approval”, “authorize”, “complete”, “gain”, “protect”, and “target” as positive words and “tank” and “sink” as negative words. We proceeded in the same way with the other dictionaries.

The basis of our selection of a suitable lexicon for the textual analysis is a correlation analysis between the sentiment scores based on the different dictionaries and the market reactions of the respective news packages, as shown in Table 1. High correlations are desirable, as they then suggest that the external information field is well proxied.

Even though some dictionaries are designed to cover a specific context, such as a financial context, it is still necessary to work with fundamentally insufficient recognizability of context by natural language processing (e.g., [44,56]). Consequently, a single message can be evaluated with a certain score, although the message is to be understood in an opposite context, depending on the perspective to be adopted. This leads to single news items being evaluated with sentiment scores that are contrary to the direction of the market reaction. For this reason, we neutralize the signs for the correlation analysis, c.f. the second row of Table 1. Since the correlation between the (news package) values neutralized by the signs and the market reactions is high, at least in two cases, we conclude that the strength B=|B| of a message can be well represented by a text analysis algorithm. The second row in Table 1 complements the first row in that it provides better insight into the extent to which B=|B| can truly be represented by the sentiment score because the correlation is not “distorted” by matching signs.

To validate our method, we draw on proven concepts in the industry. In the context of annotations by humans, an acceleration of machine learning has been achieved, which is called human-in-the-loop [67]. Similarly, we validate the automated estimation of the text analysis approach by an expert survey and adjust the “wrongly” detected signs at individual news item level for consistency. The improved consistency of the data increases the correlation at the news package level for all the dictionaries, c.f. the third row of Table 1. To calculate the parameters μ and α, however, it is critical that the signs are correct, i.e., that they match the actual direction of the message. Therefore, the validation or adjustment of the signs is performed, and the correlation is increased in all cases.

The financial dictionary of [64] is particularly suitable for our purposes from the wide range of methods, as shown by the comparatively high correlation in Table 1, regardless of whether the signs were neutralized or adjusted. The dictionary is specially designed for the analysis of financial text and is broadly used in economic research [58,68,69,70].

To assess how unambiguous a message is in each case, we use a sentiment polarity score, PS, as proposed by [71] and used similarly by [72]. Table 2 contains the measure of PS that ranges from −1 to +1 and is given by $PS=\frac{P-N}{P+N}$. The closer the score is to −1 or +1, the clearer the message is.

### 3.3. Results

Table 2 shows our sample of 18 events used to calculate μ and α. As mentioned above, six other events were identified, but these could not be considered further for the calculation because either trading in the BioNTech shares was not in dynamic equilibrium (strong changes in volatility indicated a transitional phase) or the overall market was not in dynamic equilibrium. The latter was determined in the same way as described above based on the volatilities of the leading indices (S&P 500, NASDAQ-100). To recap, the news headlines listed in the table are representative of the news packages as so-called lead headlines, which subsume other splitter news with the same statement. The entire database with 24 events, including all headlines and splitter news, is available upon request.

In Table 2, noteworthy findings include that the measured message strengths are very small, i.e., |B|≪1, and the sometimes high value of the sentiment polarity score, |PS|⪅1, indicating unique messages. The 15-min volatilities $T_{15^{\prime }}$ of BioNTech shares measured shortly before the event are of $O(10^{-3})$ and are within the 5% quantile of all volatilities measured in the observation period. The average trading potential ${\bar{N}}_{\mathrm{pot}}$ determined from the market reaction using Equation (21) correlates highly (ρ=0.87) with message B. The latter must be the requirement for a suitable experimental setting for evaluating Equation (10) in linear approximation. For k=1 USD, $T=T_{15^{\prime }}$ and B=|B| the quotient BkT is calculated in the last column. The calculated risk measures $\chi ,\;\eta ,\;c_{B}$ are suitable for describing the reaction of certain output variables to small changes in input variables in a linear approximation; see Equations (13)–(15). Assume, for example, a given volatility *T* and news situation B. The calculated susceptibility χ can then be used to estimate the market reaction if the news situation becomes slightly worse (better). In a financial report on a specific market situation, further assessments of the sensitivity of market participants could, therefore, be possible. This aspect is not pursued further here. In the analyzed events, we observe a wide range of market situations, measured by the level of sensitivity (χ, η and $c_{B}$) to external conditions, which are worth investigating in a separate line of research.

All pairs of measured values (BkT, N¯pot) in Table 2 are used to fit the curve shown in Figure 1 with Equation (9) and determine the model parameters μ and α.

Since one data point was always omitted for the calculation (jackknife resampling), 17 different estimates could be produced. The final result is the mean value over these estimates (jackknife estimators of the parameters) and is displayed in Table 3 along with the range. We refrain from calculating the variances here, as they would obscure the results, and instead present the ranges as error indicators. The parameter μ can be determined with sufficient accuracy. The parameter α, however, is difficult to estimate due to the small number of data points and a missing entry with high trading potential ${\bar{N}}_{\mathrm{pot}}$ and is therefore subject to high estimation errors. This high sensitivity is reflected in the broad bandwidth. However, a very high trading potential in the capital market can only be expected if a very strong information field B, i.e., strong news, occurs or if the volatility *T* is very low.

Considering Equations (13)–(15), it can be concluded that the risk measures χ and η can be determined with better accuracy than $c_{B}$. The risk measure $c_{B}$ can be determined less precisely because of the direct dependency on the heavily errored variable α and the error superimposition of μ and α.

### 3.4. Concept for a One-Step-Ahead-Forecast

The method presented thus far focuses on modeling the dynamics that are to be expected as a result of a new message. Thus, only sudden risk events are modeled, and the full dynamics of a share/agent system are not mapped. As a result, the following prediction concept can only make statements about the short-term price development of security that is to be expected as a result of isolated news within the framework of the statistical models.

The central equations for the prediction are Equation (21) in conjunction with Equation (9). If the parameters μ and α have been determined—or are permanently determined and updated as part of machine learning—the volatility *T* of the security must be determined continuously at the same time. A constant volatility over a period of time indicates that the share/agent system under consideration is in dynamic equilibrium. While determining the volatility, the news flow must be tracked using suitable information sources. From this, the share-specific messages are to be filtered and converted to a value B according to the method described above (Section 3.2.3). Then, x=μ|B|kT is calculated and from this the trade potential ${\bar{N}}_{\mathrm{pot}}$ according to Equation (9). Equation (21) then supplies a prediction of a new price ${\hat{P}}_{t+1}$ based on the current price $P_{t}$.

The result is a predictor for the price one-step-ahead, and this predictor is itself subject to uncertainty and obeys a probability distribution. Therefore, the predictor $\hat{P}$ serves as an indication for the price movement in the next time step. The forecast is not exact because of the underlying distribution of the predictor. Hence, risk-free excess profits cannot be achieved.

We select several benchmark approaches to evaluate the prediction results of our three-state model (3SM) in Table 4. In addition to regular prediction benchmark approaches, which have the inherent disadvantage of not being able to process the news B as information in the prediction, we also compare it with the two-state model (2SM). The regular benchmark approaches include a naive approach that uses the last value before a message as the prediction value (last observation carry forward, LOCF) and the moving average (MA) with five observations smoothing. In addition, we use an ARIMA model that is well suited for estimating the next step [73]. To incorporate a comparative model utilizing sentiment as information in prediction, we additionally estimate a regression model with ARIMA errors to account for the time series structure of the data (TS Regression). We employ the VOLQ, the volatility index of the NASDAQ 100, as an exogenous sentiment variable. Similar to the VIX, the volatility index of the S&P 500, is utilized as a sentiment measure and even for predicting short-term returns [74,75]. We leverage the VOLQ for the more narrowly focused NASDAQ 100, wherein BioNTech is included. Besides the fact that the NASDAQ 100 volatility index is better suited to predict volatility than the VIX [76], the advantage is that the VOLQ is available at the same frequency as the BioNTech data, i.e., minute-by-minute, thus allowing for better comparability than, for example, with the monthly Consumer Confidence Index. Each model is fitted with all observations up to a news item and then used to predict the next value. The error indicators used for comparability are standard and include the mean absolute error (MAE), mean squared error (MSE), mean absolute percentage error (MAPE), and root mean squared error (RMSE). Based on the prediction results, we conclude that our model is well-specified and the model parameters are well-determined, as it performs better than all benchmark methods. The Shapiro–Wilk test could not be rejected (*p*-value = 0.9265), so a normally distributed forecast error (MAPE) can be assumed with $N(0.026,\;0.032^{2})$. Thus, the forecast neither overestimates nor underestimates systematically. In addition to theoretical arguments for the use of a trinary agent system in Section 2.1.1, the comparison of the prediction performance of the two-state and three-state models provides a nuanced indication that the three-state model is marginally superior. However, despite the very similar performance of the 2SM and 3SM, the result confirms that our model calibration procedure works well. Fundamentally, it can be argued that the approaches from statistical physics have a strong advantage over regular prediction approaches in this application due to the processing of the news B.

Depending on the new price, investor-internal reports can then be written and/or micro hedges initiated. In the case of a machine learning implementation with a continuous learning algorithm and a suitable loss function [77], the process could be improved, fully automated, and operate in subminute time ranges.

## 4. Conclusions

In recent decades, increasing numbers of models have been used in econometrics that have been adopted from statistical physics. The development is well advanced, and complex theoretical models are the subject of many simulative and numerical investigations. With all models, there is always the question of how to determine the model parameters. In the case of models applied to securities, in particular, the concern is how the parameters can be estimated on the basis of capital market data. We investigated this question and examined the basic model of an ideal agent system in greater detail, and developed a procedure for parameter estimation. The more complex models mentioned above, which incorporate social interactions between agents, are based on this basic model, so the methods developed for parameter estimation are also useable in advanced model approaches. In our analysis, we adhered to the extension of the commonly used binary agent system to include the hold position to a trinary agent system and derived corresponding equations to describe the dynamics, which serve as a starting point for parameter estimation. In physics, special experiments are designed to determine model parameters and to research phenomena of interest. In the field of finance, it will be difficult to construct an experiment under laboratory conditions to implement a similar approach. Here one is dependent on the abundance and correct use of data from the capital market. The art of the financial experimenter is to find the data that most closely resemble the phenomenon under study and then perform the modeling based on those data. Cut-out experiments must, therefore, be defined in which the specific research question can be analyzed. For the case of the ideal agent system with the three states “sell”, “hold”, and “buy”, we have shown a modeling approach and paid special attention to the suitable selection of the central message for an event influencing the price of a share. In addition, we have derived and reported key risk indicators that characterize the sensitivity of the system at a specific operating point. We also examined the question of a possible forecast concept and found a way to describe the price movement triggered by a message as part of a short-term forecast. The performance comparison with selected benchmark approaches shows that our calibrated three-state model provides better predictions than regular benchmark approaches. In addition, we found nuanced advantages of the three-state model over the two-state model, which we used as another benchmark approach. Therefore, it can be concluded in principle that approaches from statistical physics have a strong advantage over regular prediction approaches due to the processing of sudden news. The use of algorithms from the field of machine learning could map the presented method and thus generate warnings or risk reports in less than a minute in the event of a risk or suggest microhedge strategies and cope with the shock of the sudden event. In connection with this, the approach chosen here must be evaluated critically since various manual interventions had to be accepted for the sake of more consistent results. Thus, our application mainly identifies limitations in the analysis of the news situation and can be further improved in this direction. These improvements depend, for example, on further developments in the field of linguistic text analysis, in particular to more precisely and reliably recognize contexts and valences. However, the results indicate that our calibration procedure works well and that the model was correctly specified, and the model parameters were well determined. In a research project based on the results presented here, the question of a suitable calibration of generalized agent systems with coupled dynamic components can be addressed and a procedure can be designed how, on the basis of capital market data from special market phases, the required coupling parameter can be estimated. Then, the complete set of model parameters of the generalized agent system is available.

## Figures and Tables

**Table 1:** Correlation analysis between sentiment scores based on different dictionaries and market reactions (*p*-values in parentheses).

	Loughran-McDonald	Henry	Harvard-IV	QDAP
sign unmodified	+0.31 $\left(2.14\times 10^{-1}\right)$	+0.52 $\left(2.64\times 10^{-2}\right)$	+0.26 $\left(2.93\times 10^{-1}\right)$	+0.10 $\left(6.93\times 10^{-1}\right)$
sign neutralized	+0.58 $\left(1.10\times 10^{-2}\right)$	−0.07 $\left(7.97\times 10^{-1}\right)$	−0.07 $\left(7.88\times 10^{-1}\right)$	+0.44 $\left(6.82\times 10^{-2}\right)$
sign adjusted	+0.87 $\left(2.36\times 10^{-6}\right)$	+0.67 $\left(2.17\times 10^{-3}\right)$	+0.68 $\left(1.85\times 10^{-3}\right)$	+0.80 $\left(7.73\times 10^{-5}\right)$

**Table 2:** Analyzed news and deduced key figures (k=1 USD).

Date	*Core Message*							
Analysis	News		Capital Market		Risk-Measures			
	B	PS	$T_{15^{\prime }}$	${\bar{N}}_{\mathrm{pot}}$	χ	η	$c_{B}$	BkT
Units	$10^{-2}$	$10^{-1}$	$10^{-4}$	$10^{-3}$	$10^{-2}$	$10^{-1}$	$10^{-5}$	$10^{0}$
							USD	
3 August 2021	*F.D.A. Aims to Give Final Approval to Pfizer Vaccine by Early Next Month*							
	+6	+7	+10	+11	+26	−161	−46	+62
6 August 2021	*FDA expects to have COVID vaccine booster strategy early next month*							
	0	NA	+11	+22	+20	−260	−140	+160
20 August 2021	*FDA Approves Pfizer-Biontech COVID-19 Vaccine*							
	+14	+10	+13	+31	+20	−225	−149	+111
10 September 2021	*Biontech to Seek Vaccine Approval for 5–11 year olds*							
	+8	+6	+9	+11	+28	−235	−86	+84
13 September 2021	*Covid Evidence doesn’t support broad Need for Boosters*							
	−3	−10	+12	−8	−22	+51	−7	−24
16 September 2021	*FDA Advisers Back a Narrower Authorization for Pfizer Booster*							
	−10	−6	+13	−9	−20	+157	−73	−77
23 September 2021	*FDA Authorizes Booster Dose of Pfizer-BioNTech COVID-19 Vaccine for*							
	*Certain Populations*							
	+8	+10	+10	+8	+25	−194	−73	+78
30 September 2021	*Pfizer/BioNTech Vaccine Antibodies Disappear*							
	−3	−3	+14	−42	−18	+43	−7	−24
18 October 2021	*Pfizer And AstraZeneca Vaccines Were Effective As Prior Infection,*							
	*U.K. Study Finds*							
	+12	+10	+8	+23	+34	−510	−273	+150
20 October 2021	*CDC: Pfizer COVID-19 Vaccine Highly Protective in 12–18 Age Group*							
	+6	+5	+7	+14	+40	−390	−117	+98
4 November 2021	*U.K. Regulator Is First to Approve Merck’s COVID-19 Pill*							
	−13	−10	+9	−26	−29	+407	−247	−143
5 November 2021	*Update 2: Pfizer says antiviral pill cuts risk of severe COVID-19 by 89%*							
	−18	−10	+13	−63	−21	+293	−240	−141
26 November 2021	*Vaccine Stocks Jump Premarket Amid New Variant Fears, EU Backing*							
	+19	+10	+12	+56	+21	−324	−282	+152
6 December 2021	*Vaccine Stocks Slip as Street Weighs Omicron Variant Uncertainty*							
	−14	−10	+12	−38	−21	+243	−162	−115
10 December 2021	*Researchers in South Africa have also found a drop-off in the level of antibody*							
	*protection from that vaccine versus the new strain*							
	−8	−5	+17	−35	−16	+76	−28	−48
31 December 2021	*Pfizer Vaccine Causes Myocarditis*							
	−8	−10	+12	−4	−22	+138	−49	−64
10 January 2022	*Pfizer CEO: Developing Omicron Targeted Vaccine*							
	+9	+10	+105	+3	+2	−2	−1	+9
21 January 2022	*Less-Threatening Omicron Lowers Covid Vaccine Sales Estimate*							
	−10	−10	+20	−13	−13	+62	−28	−48

**Table 3:** Results for μ and α along with the range of variation from the estimation.

Parameter	Value	Range
μ ($10^{-4}$ USD)	3.92	(3.51–4.21)
α	0.75	(0.00–1.00)

**Table 4:** Results of an (in-sample) one-step-ahead forecast using the 18 events from Table 2 with benchmark approaches and four error indicators.

	MAE	MSE	MAPE	RMSE
3SM	7.707	86.987	0.026	9.327
2SM	7.707	87.009	0.026	9.328
LOCF	11.079	217.087	0.036	14.734
MA	11.688	221.481	0.038	14.882
ARIMA	11.064	216.700	0.036	14.721
TS Regression	11.103	218.361	0.036	14.777

## Data Availability

Data are available upon request.

## Appendix A. Table of Correspondence

**Table A1:** Correspondence of physical model parameters in an econometric context, substantiated with relevant literature.

Model Parameters			Econometrics	Literature
**External** **Parameters**	B	Magnetic Field	News Sentiment	*Public Information that Affects all Agents; Investment Environment; Preference Parameter:* [15,19,23,24,25,26]
	*T*	Temperature	Volatility	*Noise; Irrationality; Degree of Randomness in Agents’ Decisions; Collective Climate Parameter or Volatility:* [15,19,23,27,29,31,52,53]
	*N*	Number of Particles	Number of Shares	*Number of Traders in buying/selling Positions:* [30]
**Model** **Parameters**	μ	Magnetic Moment	Willingness to Trade	*Willingness to adopt/buy; Idiosyncratic Judgment:* [19,24,25,26,31]
	*k*	Boltzmann Constant	Scale/Unit Parameter	Unspecified in Literature (model endogenous)—mediates between Entropy and Energy.
	α	Energy of State $n_{0}$	Energy Share of “hold”-Position	
**Target** **Parameters**	*E*	Energy	Investors’ “Utility”	*Utility Function:* [19]
	*S*	Entropy	Entropy	*Measure of Uncertainty:* [34,48]
	*M*	Magnetization	Purchase/Sale Potential	*Aggregate Demand; Average Opinion; Net-Demand:* [19,24,28,30]
	$N_{\mathrm{pot}}$	−	Trade Potential	*Active Agents:* [32]
	χ	Susceptibility	Overall System Sensitivity	*Depth parameter of the market which measures sensitivity of price fluctuation in response to changes in excess demand:* [30]
	η	Thermal loss Coefficient	Overall System Sensitivity	Unspecified in Literature. Parameter which measures sensitivity of trade potential in response to changes in volatility
	$c_{B}$	Capacity	Overall SystemSensitivity	Unspecified in Literature. Parameter which measures sensitivity of utility in response to changes in volatility

## References

[1] Föllmer H. (1974). Random economies with many interacting agents. J. Math. Econ..

[2] Cont R., Tankov P. (2004). Financial Modelling with Jump Processes.

[3] Sornette D. (2014). Physics and financial economics (1776–2014): Puzzles, Ising and agent-based models. Rep. Prog. Phys..

[4] Werker C., Brenner T. (2004). Empirical calibration of simulation models. Pap. Econ. Evol..

[5] LeBaron B., Tesfatsion L., Judd K.L. (2006). Chapter 24 Agent-based Computational Finance. Handbook of Computational Economics.

[6] Windrum P., Fagiolo G., Moneta A. (2007). Empirical Validation of Agent-Based Models: Alternatives and Prospects. J. Artif. Soc. Soc. Simulaion.

[7] Fagiolo G., Moneta A., Windrum P. (2007). A Critical Guide to Empirical Validation of Agent-Based Models in Economics: Methodologies, Procedures, and Open Problems. Comput. Econ..

[8] Chen S.H., Chang C.L., Du Y.R. (2012). Agent-based economic models and econometrics. Knowl. Eng. Rev..

[9] Iori G., Porter J. (2012). Agent-Based Modelling for Financial Markets.

[10] Fagiolo G., Guerini M., Lamperti F., Moneta A., Roventini A., Beisbart C., Saam N.J. (2019). Validation of Agent-Based Models in Economics and Finance. Computer Simulation Validation: Fundamental Concepts, Methodological Frameworks, and Philosophical Perspectives.

[11] Isihara A. (1971). Statistical Physics.

[12] Landau L.D., Lifšic E.M. (1980). Course of Theoretical Physics.

[13] Greiner W., Neise L., Stöcker H. (1995). Thermodynamics and Statistical Mechanics.

[14] Kardar M. (2007). Statistical Physics of Particles.

[15] Weidlich W. (1971). The Statistical Description of Polarization Phenomena in Society. Br. J. Math. Stat. Psychol..

[16] Galam S., Gefen Y., Shapir Y. (1982). Sociophysics: A new approach of sociological collective behaviour. I. Mean–behaviour description of a strike. J. Math. Sociol..

[17] Chakraborti A., Toke I.M., Patriarca M., Abergel F. (2011). Econophysics review: I. Empirical facts. Quant. Financ..

[18] Chakraborti A., Toke I.M., Patriarca M., Abergel F. (2011). Econophysics review: II. Agent-based models. Quant. Financ..

[19] Bouchaud J.P. (2013). Crises and Collective Socio-Economic Phenomena: Simple Models and Challenges. J. Stat. Phys..

[20] Schinckus C. (2016). 1996–2016: Two decades of econophysics: Between methodological diversification and conceptual coherence. Eur. Phys. J. Spec. Top..

[21] Schinckus C. (2018). Ising model, econophysics and analogies. Phys. A Stat. Mech. Its Appl..

[22] Kutner R., Ausloos M., Grech D., Di Matteo T., Schinckus C., Eugene Stanley H. (2019). Econophysics and sociophysics: Their milestones & challenges. Phys. A Stat. Mech. Its Appl..

[23] Kaizoji T. (2000). Speculative bubbles and crashes in stock markets: An interacting-agent model of speculative activity. Phys. A Stat. Mech. Its Appl..

[24] Michard Q., Bouchaud J.P. (2005). Theory of collective opinion shifts: From smooth trends to abrupt swings. Eur. Phys. J. B.

[25] Sornette D., Zhou W.X. (2006). Importance of positive feedbacks and overconfidence in a self-fulfilling Ising model of financial markets. Phys. A Stat. Mech. Its Appl..

[26] Borghesi C., Bouchaud J.P. (2007). Of songs and men: A model for multiple choice with herding. Qual. Quant..

[27] Oh W., Jeon S. (2007). Membership Herding and Network Stability in the Open Source Community: The Ising Perspective. Manag. Sci..

[28] Vikram S.V., Sinha S. (2011). Emergence of universal scaling in financial markets from mean-field dynamics. Phys. Rev. E.

[29] Krause S.M., Bornholdt S. (2012). Opinion formation model for markets with a social temperature and fear. Phys. Rev. E.

[30] Zhang B., Wang J., Fang W. (2015). Volatility behavior of visibility graph EMD financial time series from Ising interacting system. Phys. A Stat. Mech. Its Appl..

[31] Crescimanna V., Di Persio L. (2016). Herd Behavior and Financial Crashes: An Interacting Particle System Approach. J. Math..

[32] Fernandez M.A., Korutcheva E., de La Rubia F.J. (2016). A 3-states magnetic model of binary decisions in sociophysics. Phys. A Stat. Mech. Its Appl..

[33] Foley D.K. Statistical Equilibrium in Economics: Method, Interpretation, and an Example. Proceedings of the XII Workshop on General Equilibrium: Problems, Prospects and Alternatives.

[34] Marsili M. (1999). On the multinomial logit model. Phys. A Stat. Mech. Its Appl..

[35] Iori G. (1999). Avalanche Dynamics and Trading Friction Effects on Stock Market Returns. Int. J. Mod. Phys. C.

[36] Cont R., Bouchaud J.P. (2000). Herd Behavior and Aggregate Fluctuations in Financial Markets. Macroecon. Dyn..

[37] Takaishi T. (2005). Simulations of financial markets in a Potts-like model. Int. J. Mod. Phys. C.

[38] Sato A.H. (2007). Frequency analysis of tick quotes on the foreign exchange market and agent-based modeling: A spectral distance approach. Phys. A Stat. Mech. Its Appl..

[39] Takaishi T. (2013). Analysis of Spin Financial Market by GARCH Model. J. Phys. Conf. Ser..

[40] Anderson S.P., de Palma A., Thisse J.F. (2001). Discrete Choice Theory of Product Differentiation.

[41] Bouchaud J.P. (2009). The (unfortunate) complexity of the economy. Phys. World.

[42] Sun L., Najand M., Shen J. (2016). Stock return predictability and investor sentiment: A high-frequency perspective. J. Bank. Financ..

[43] Gao B., Yang C. (2017). Forecasting stock index futures returns with mixed-frequency sentiment. Int. Rev. Econ. Financ..

[44] Renault T. (2017). Intraday online investor sentiment and return patterns in the U.S. stock market. J. Bank. Financ..

[45] Pan W.F. (2020). Does Investor Sentiment Drive Stock Market Bubbles? Beware of Excessive Optimism!. J. Behav. Financ..

[46] Weiss P. (1907). L’hypothèse du champ moléculaire et la propriété ferromagnétique. J. Phys. Théorique Appl..

[47] Castellano C., Fortunato S., Loreto V. (2009). Statistical physics of social dynamics. Rev. Mod. Phys..

[48] Shannon C.E. (1948). A Mathematical Theory of Communication. Bell Syst. Tech. J..

[49] Jaynes E.T. (1957). Information Theory and Statistical Mechanics. Phys. Rev..

[50] Brusentsov N.P., Alvarez J.R., Impagliazzo J., Proydakov E. (2011). Ternary Computers: The Setun and the Setun 70.

[51] Nadal J.P., Chenevez O., Weisbuch G., Kirman A. (1998). A Formal Approach to Market Organization: Choice Functions, Mean Field Approximation and Maximum Entropy Principle. Proceedings of the Self-Organization and Evolutionary Economics: New Developments, CNAM, Paris, France, 30 September–1 October 1996.

[52] de Mattos Neto P.S.G., Silva D.A., Ferreira T.A.E., Cavalcanti G.D.C. (2011). Market volatility modeling for short time window. Phys. A Stat. Mech. Its Appl..

[53] Boerner C.J., Hoffmann I., Stiebel J.H. (2023). On the connection between temperature and volatility in ideal agent systems. J. Stat. Mech. Theory Exp..

[54] Boerner C.J., Hoffmann I., Stiebel J.H. (2023). Generalized Agent System with Triplet States: Model Parameter Identification of Agent-Agent Interaction. SSRN Electron. J..

[55] Kearney C., Liu S. (2014). Textual sentiment in finance: A survey of methods and models. Int. Rev. Financ. Anal..

[56] Loughran T., McDonald B. (2016). Textual Analysis in Accounting and Finance: A Survey. J. Account. Res..

[57] Loughran T., McDonald B. (2020). Textual Analysis in Finance. Annu. Rev. Financ. Econ..

[58] Stangor P., Kuerzinger L. (2021). Measuring investor sentiment from Social Media Data—An emotional approach. SSRN Electron. J..

[59] Feinerer I., Hornik K., Meyer D. (2008). Text Mining Infrastructure in R. J. Stat. Softw..

[60] Mechura M.B. Lemmatization List: English (en) [Data File]. http://www.lexiconista.com.

[61] Rinker T.W. (2018). textstem: Tools for Stemming and Lemmatizing Text. Version 0.1.4. https://CRAN.R-project.org/package=textstem.

[62] Feuerriegel S., Proellochs N. (2023). SentimentAnalysis: Dictionary-Based Sentiment Analysis. Version 1.3-5. https://CRAN.R-project.org/package=SentimentAnalysis.

[63] Henry E. (2008). Are Investors Influenced By How Earnings Press Releases Are Written?. J. Bus. Commun..

[64] Loughran T., McDonald B. (2011). When is a liability not a liability? Textual analysis, dictionaries, and 10-Ks. J. Financ..

[65] Rinker T.W. (2013). qdapDictionaries: Dictionaries to Accompany the qdap Package. Version 1.0.7. https://CRAN.R-project.org/package=qdapDictionaries.

[66] Kawasaki Z., Nirenburg S. (1993). Organizational Use of Machine Translation Systems. Progress in Machine Translation.

[67] Wu X., Xiao L., Sun Y., Zhang J., Ma T., He L. (2021). A Survey of Human-in-the-loop for Machine Learning. arXiv.

[68] Chen H., De P., Hu Y., Hwang B.H. (2014). Wisdom of Crowds: The Value of Stock Opinions Transmitted through Social Media. Rev. Financ. Stud..

[69] Da Z., Engelberg J., Gao P. (2015). The Sum of All FEARS Investor Sentiment and Asset Prices. Rev. Financ. Stud..

[70] Löffler G., Norden L., Rieber A. (2021). Negative news and the stock market impact of tone in rating reports. J. Bank. Financ..

[71] Zhang W., Skiena S. Trading strategies to exploit blog and news sentiment. Proceedings of the Fourth International aAAI Conference on Weblogs and Social Media.

[72] Li X., Xie H., Chen L., Wang J., Deng X. (2014). News impact on stock price return via sentiment analysis. Knowl.-Based Syst..

[73] Siami-Namini S., Tavakoli N., Siami Namin A. (2019). A Comparative Analysis of Forecasting Financial Time Series Using ARIMA, LSTM, and BiLSTM. arXiv.

[74] Feldman T. (2010). A More Predictive Index of Market Sentiment. J. Behav. Financ..

[75] Ding W., Mazouz K., Wang Q. (2021). Volatility timing, sentiment, and the short-term profitability of VIX-based cross-sectional trading strategies. J. Empir. Financ..

[76] Corrado C.J., Miller J.T.W. (2005). The forecast quality of CBOE implied volatility indexes. J. Futur. Mark..

[77] Goodfellow I., Bengio Y., Courville A. (2016). Deep learning. Adaptive Computation and Machine Learning.

